# A Combination of 3D-QSAR, Molecular Docking and Molecular Dynamics Simulation Studies of Benzimidazole-Quinolinone Derivatives as iNOS Inhibitors

**DOI:** 10.3390/ijms130911210

**Published:** 2012-09-10

**Authors:** Hao Zhang, Jinhang Zan, Guangyun Yu, Ming Jiang, Peixun Liu

**Affiliations:** Key Lab of Tianjin Molecular Nuclear Medicine, Institute of Radiation Medicine, Peking Union Medical College, Chinese Academy of Medical Sciences, Tianjin 300192, China; E-Mails: zhanghao27@126.com (H.Z.); sinokang123@yahoo.com.cn (J.Z.); yuguangyun123.good@163.com (G.Y.); jiangming_159@yahoo.com.cn (M.J.)

**Keywords:** 3D-QSAR, benzimidazole-quinolinone derivatives, molecular dynamics simulation, molecular docking, iNOS inhibitor

## Abstract

Inducible Nitric Oxide Synthase (iNOS) has been involved in a variety of diseases, and thus it is interesting to discover and optimize new iNOS inhibitors. In previous studies, a series of benzimidazole-quinolinone derivatives with high inhibitory activity against human iNOS were discovered. In this work, three-dimensional quantitative structure-activity relationships (3D-QSAR), molecular docking and molecular dynamics (MD) simulation approaches were applied to investigate the functionalities of active molecular interaction between these active ligands and iNOS. A QSAR model with *R*^2^ of 0.9356, *Q*^2^ of 0.8373 and Pearson-*R* value of 0.9406 was constructed, which presents a good predictive ability in both internal and external validation. Furthermore, a combined analysis incorporating the obtained model and the MD results indicates: (1) compounds with the proper-size hydrophobic substituents at position 3 in ring-C (R_3_ substituent), hydrophilic substituents near the X_6_ of ring-D and hydrophilic or H-bond acceptor groups at position 2 in ring-B show enhanced biological activities; (2) Met368, Trp366, Gly365, Tyr367, Phe363, Pro344, Gln257, Val346, Asn364, Met349, Thr370, Glu371 and Tyr485 are key amino acids in the active pocket, and activities of iNOS inhibitors are consistent with their capability to alter the position of these important residues, especially Glu371 and Thr370. The results provide a set of useful guidelines for the rational design of novel iNOS inhibitors.

## 1. Introduction

In the past decades, nitric oxide has been conceived as a signaling and effecter molecule. It has been demonstrated that NO can be produced by three subtypes of the nitric oxide synthases (NOSs) [1]. Type I NOS (neuronal NOS, nNOS) plays a role in skeletal muscle relaxation and cerebral blood flow [2,3], and type III NOS (endothelial NOS, eNOS) relaxes smooth muscle in the vasculature through activation of cGMP [4,5]. Type II NOS (inducible NOS, iNOS) was first described in macrophages as a mechanism of macrophage cytotoxicity, and previous studies reveal that it is expressed and activated during inflammatory events [6–8]. Generally, this isoform is not expressed in healthy quiescent cells and NO is sustained at a low level. However, when iNOS is stimulated by various inflammatory stimuli (proinflammatory cytokines or lipopolysaccharide), a high level of NO is produced and it induces tissue injury at the inflammatory site [9,10]. Considerable evidence has shown that overproduction of NO induced by iNOS has been implicated in various pathological diseases including septic shock, tissue damage, and rheumatoid arthritis (RA) [11,12]. Therefore, iNOS has become a potential target for drug development in the treatment of inflammatory diseases.

Meanwhile, consistent progress has been made in discovering more drug-like inhibitors of iNOS. However, there is still some room for potent and selective inhibitors of iNOS that would be more drug-like and suitable for progression to the market as drugs [13–15]. The difficulties lie in achieving isoform specificity, targeting to specific cells or tissues and ensuring that the correct degree of inhibition is achieved [16,17]. Meanwhile, an alternative approach is to block enzyme dimerization [18]. In fact, the dimerization inhibitors have been proved to possess potentially beneficial effects in the usual screens of endotoxin-induced nitrate formation and adjuvant arthritis, and also in experimental allergic encephalomyelitis as a model of demyelinating disease [19,20]. Recently Joseph *et al.* developed a novel series of benzimidazole-quinolinone iNOS inhibitors with low clearance and sustained exposure *in vivo* [21]. This series of compounds were identified as potent iNOS selective or dual iNOS/nNOS inhibitors with selectivity over eNOS. At the same time, they also had high-efficient pharmacokinetics and suitable drug properties for development as neuropathic pain therapeuticals. However, the interaction between iNOS and ligand is not understood completely, and the related mechanism is not clear. In this paper, we report a 3D-QSAR analysis of this series of iNOS inhibitors. The large variations in binding affinities of these compounds with iNOS and the relation between biological activity and the flap motion of the enzyme, as well as, the connection between the biological activity and the conformational changes in the catalytic site of the iNOS, were investigated using a mixed approach including docking and molecular dynamics simulations. The following two steps in our computational strategy were adopted: (i) In order to construct 3D-QSAR comparative molecular similarity indices analysis (atom-based 3D-QSAR model) models, we used 39 known iNOS inhibitors whose activities had been experimentally reported (Table 1) [21]; (ii) In order to explore proper coordinates of the iNOS/benzimidazole-quinolinone inhibitors complex in docking as well as to understand the reason for the large variations in the binding affinities of the inhibitors with iNOS, molecular dynamics (MD) simulation was employed. It was found that results from MD were highly consistent with the findings obtained from the atom-based 3D-QSAR model.

## 2. Results and Discussion

### 2.1. 3D-QSAR Analyses

**Figure 1:**
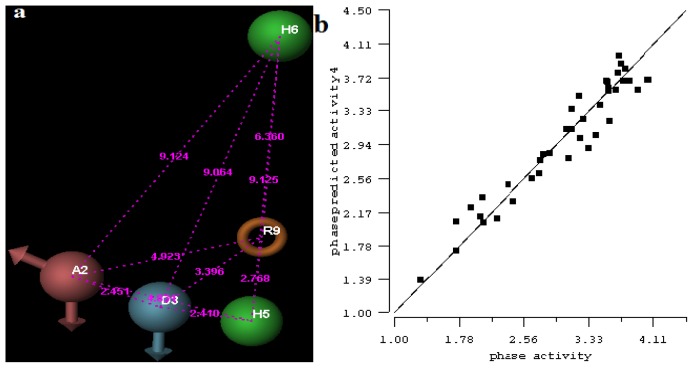
(**a**) Common pharmacophore for active ligands. Pharmacophore features are color-coded: dark blue H-donor, brown H-acceptor, filemot aromatic ring, green hydrophobic group. All distances between pharmacophore features are reported in Ångstroms; (**b**) Fitness graph between observed activity and Phase predicted activity for training and test set compounds.

All pharmacophore hypotheses produced in the step of searching for common pharmacophores were used to build 3D-QSAR models. After analyzing the alignment between the active ligands and the generated hypothesis, a best hypothesis ADHHR.89 was selected for further research. The selected hypothesis contained one hydrogen bond donor, one hydrogen bond acceptor, one aromatic ring and two hydrophobic groups, as shown in Figure 1a. The QSAR model generated by hypothesis ADHHR.89 was constructed by an atom-based QSAR modeling approach, of which a molecule was treated as a set of overlapping van der Waals spheres. The atom-based QSAR modeling approach was applicable to the structures with a relatively small number of rotatable bonds and common structural framework [22,23]. Atom-Based QSAR models were created by applying partial least squares (PLS) regression to a large set of binary-valued variables that encode whether or not ligand atoms occupy various cube-shaped elements of space [24]. The accuracy of the models improved with the increasing number of PLS factors until overfitting starts to occur. In this study, the best QSAR model with 4 PLS factors was employed, and the QSAR visualization can be shown by cubic 3D grids mapped by ligand atoms. A report suggested that Phase performed well when *Q*^2^ > 0.7 or *R*^2^ > 0.4 [25]. The statistical parameters associated in the generated QSAR model were as follows: The training set achieved *R*^2^ (value of *R*^2^ for the regression) of 0.9356, with an SD (standard deviation of the regression) of 0.2863; the test set obtained *Q*^2^ (value of *Q*^2^ for the predicted activities) of 0.8373, with an RMSE (ROOT-mean-square error) of 0.2567; and Pearson-R (Pearson *R* value for the correlation between the predicted and observed activity for the test set) of 0.9406. The *p* value of 1.643 × 10^−14^ indicated a high degree of confidence. The regression line for the observed and Phase predicted activity was shown in Figure 1b. The predicted activities of the training and test set molecules were also listed in Table 1.

**Figure 2:**
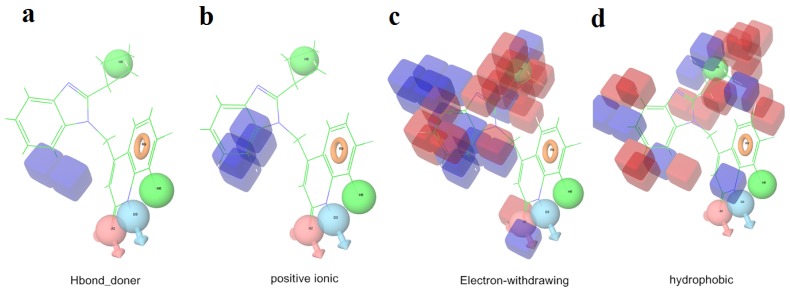
Pictorial representation of the contours generated using the quantitative structure-activity relationships (QSAR) model. Hydrogen bond acceptor property and electron withdrawing features that arise when the QSAR model is applied to compounds 15 (**a**) and (**c**). Positive ionic and hydrophobic interactions features that arise when the QSAR model is applied to compounds 15 (**b**) and (**d**). Blue cubes favorable regions for activity, red cubes unfavorable region for activity.

The 3D-QSAR visualization can be generated by Phase, in which the blue cubes are favorable for activity and the red cubes are unfavorable. It could be concluded from Figure 2 that the heterocyclic ring-D may improve a compound’s activity because of the blue and red cubes observed at the ring-D. The corresponding compounds with heterocyclic ring-D (compounds 26, 34, 37, 38) are more active than compounds with aromatic ring-D (compounds 15, 18). Furthermore, presence of hydrophilic grouping around the 4-position of ring-D would enhance the iNOS inhibition according to Figure 2d. The structures of ligands 26 and 32 are identical except for the 7 position, while the activity of ligand 32 is interesting due to *N* in the 7 position. The red cubes at position 7 in ring-D indicated a positive potential of electron withdrawing, characteristic of the ligands from Figure 2c. For example, the activities of compounds 27, 28, 29, 30 were lower than compounds 31. The R_3_ in ring-C substituents of the 39 ligands was found not to be electron-withdrawing and quite sensitive to steric bulk from Figure 2c,d, such as an ethyl group (compound 11; pEC_50_ = 2.37) or expansion to a tertiary butyl group (compound 12; pEC_50_ = 1.32) which led to significant decreases in potency. However, the simple n-propyl substituent maintained activity (compound 13; pEC_50_ = 3.14). Cyclization of the isopropyl group to afford the cyclo-propyl derivative, compound 14, led to a minor loss in potency (compound 14; pEC_50_ = 2.80). However, upon expansion of the ring size to cyclo-butyl (compound 15; pEC_50_ = 3.59) or cyclo-pentyl (compound 16; pEC_50_ = 3.13), potency improved. Further increase in ring size like cyclo-hexyl of compound 17 led to a loss in potency (compound 17; pEC_50_ = 2.05), and thus demonstrated subtle R_3_ size requirements for optimal potency.

### 2.2. Docking Results

**Figure 3:**
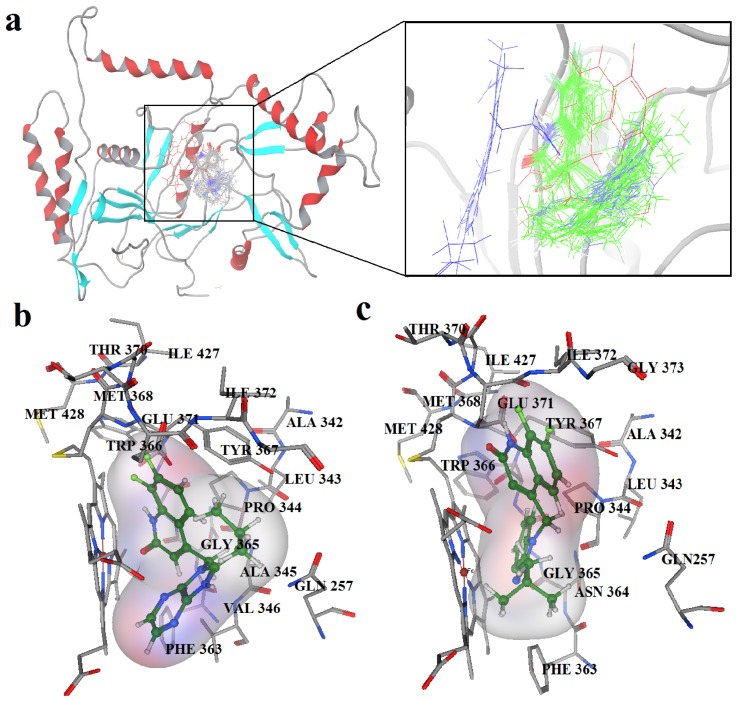
(**a**) Docked structures of all 39 inhibitors; (**b**) The binding site formed around compound 34; (**c**) The binding site formed around compound 12.

Figure 3a showed that all 39 inhibitors were docked into the binding pocket of iNOS. All molecules were positioned in the same way, and most of them shared a similar binding mode except for several molecules deviating from the atom Fe due to low activity with unfavorable features for the binding process. The binding modes of the most active compound, compound 34, and compound 12 with the least activity are shown in panels b and c of Figure 3, respectively.

Accordingly, compound 34 occupied the binding pocket, and the its quinolinone ring coordinated to the Fe and interacted with Tyr367. Compared with the crystal structure of BBS-4 bound to iNOS (PDB code: 2ORO), compound 34 performed an identical binding mode whereas compound 12 adopted a distinct conformation in the pocket compared with most compounds. This difference in the binding modes resulted in distinct activities. The detailed interactions will be discussed further in the following molecular dynamics’ simulations.

### 2.3. Molecular Dynamics’ Simulation Studies

**Figure 4:**
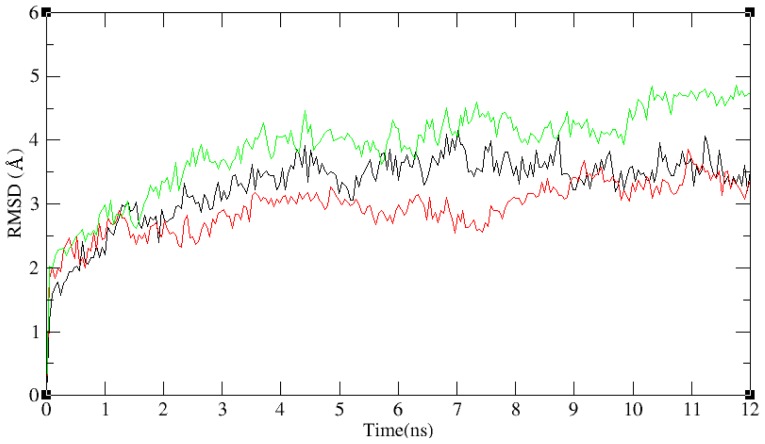
The results of molecular dynamics (MD) simulation. The MD simulation time *vs*. root mean-square deviation (RMSD, in Å) of the backbone atoms for the system-I (green), the 34-bound system (black) and the 12-bound system (red).

In the docking studies, flexibility of the protein was not taken into consideration. In order to confirm binding modes of ligands and to give the whole impression of the benzimidazole-quinolinone derivatives, we performed MD simulations with the Desmond program. On the whole, the coordinates of active site residues of inhibitors bound to iNOS may be different to the coordinates of free iNOS. For this reason, two different systems were used in our MD simulations: The first one was the iNOS and solvent (water) molecules (system-I), and the second was a rectangular box which included benzimidazole-quinolinone analogues in the binding site of the enzyme (system-II). System-I was run as a control, and compound 34 as the most potent compound, and compound 12 with low activity in Table 1 were docked in the binding site of iNOS. The 12-bound system and the 34-bound system consisted of system-II. To explore the dynamic stability of systems and ensure the rationality of the sampling method, RMSD from the starting structure was analyzed as depicted in Figure 4. After 10 ns, the RMSD of system-I and system-II was about 4.5 Å and 3 Å respectively, and both of them almost remained at their own levels in the following simulation process. This indicated that the complex structure is stable after 10 ns of simulation. Average conformations of system-I and II were derived from the last 160 conformations in the last 2 ns MD simulations.

**Figure 5:**
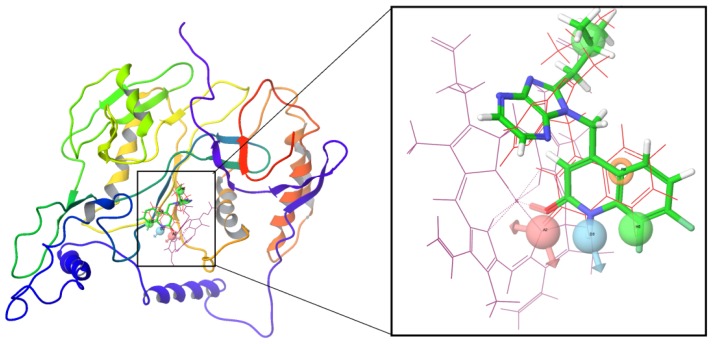
Superposition of conformation of compound 34 after MD simulation (red) and compound 34’s pose of the ADHHR.89 model (green).

**Figure 6:**
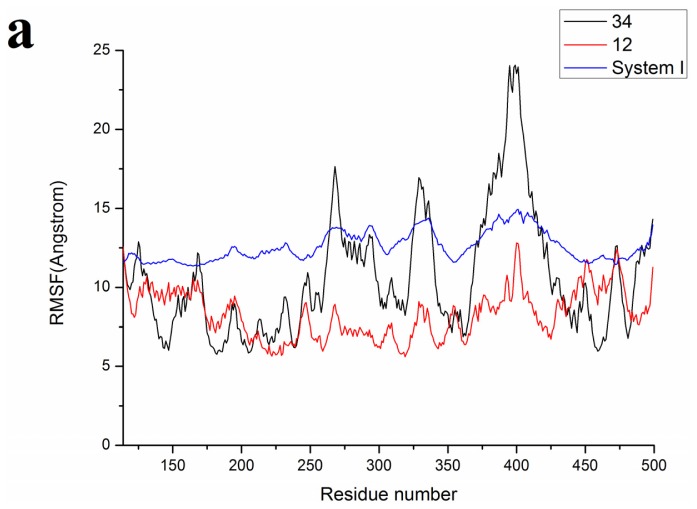

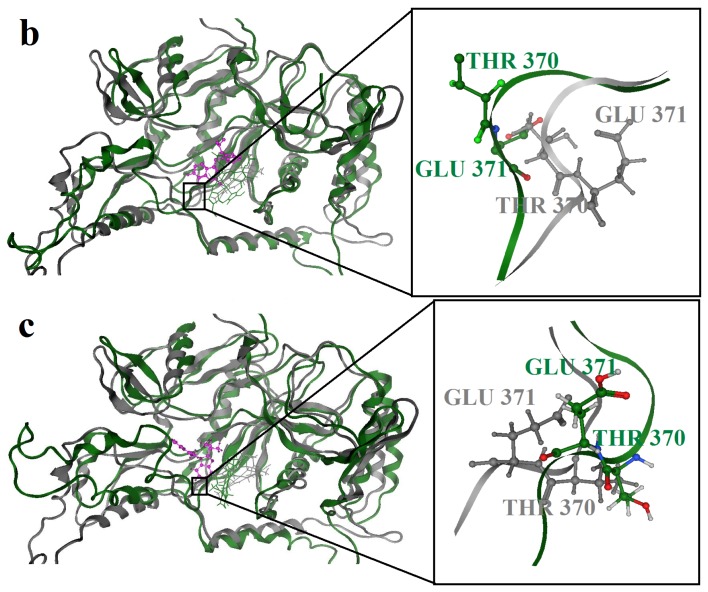
(**a**) RMSF of the protein inducible Nitric Oxide Synthase (iNOS) atom for each residue for the system-I, 34-bound and 12-bound iNOS systems. The average structure of both systems I and II from MD simulations have been compared in order to understand which parts in the active site of the enzyme are more stable and which are floppy; (**b**) the systems I (gray) and the 34-bound system (green); (**c**) the systems I (gray) and the 12-bound system (green).

Compared with molecular docking results, MD simulation results of compound 34 showed a similar binding mode, lending credit to the reliability of active conformations obtained by Glide. Besides, the mode of the most potent inhibitors, 34 resembles the 34’s pose of the ADHHR.89 model as shown in Figure 5. David *et al.* proposed that there is a direct correlation between inhibitory capability of a compound and the folding of a helix [15]. Their study revealed that inhibitors occupied the Glu371 which is part of the helix, thus displacing helix side chains from the Arg binding site and disrupting part of the dimer interface, finally blocking the formation of the protein-protein interaction present in the dimeric form of iNOS [8,10]. In view of these facts, the analysis of root-mean-square fluctuation (RMSF) *versus* the residue number for system-I and system-II is illustrated in panel a of Figure 6. It can be seen that there are four major flexible protein segments corresponding to residues 264–272, 327–337, 370–380, 389–409 in system-II. The fluctuations of these residues are higher in the 34-bound system than those in the 12-bound system. The active site, including Met368, Trp366, Gly365, Tyr367, Phe363, Pro344, Gln257, Val346, Asn364, Met349, Thr370, Glu371 and Tyr485, also have larger conformational drift for the 34-bound system than those for the 12-bound system. Compared with system-II, the protein structure of system-I changed equably and the fluctuations of the residues are small. Besides, the RMSF of residues in the active site also seemed relatively wavy, which could be caused by movement of the heme during MD simulation in view of its particular structure and function in iNOS.

In Figure 6b and c, average conformation of system-I is superimposed on that of the 12-bound system and the 34-bound system respectively. It can be observed that both 12 and 34 alter the conformation of the protein which agrees with the result of RMSF, and we found that only a few perturbations around the initial distance were found in the distance between the Fe atom and most of the residues in the active pocket. However, to the 34-bound system, the residue Glu371 and Thr370’s position changed a lot compared to the system-I’s result. On the contrary, there was little variation in the position of the same residues in the 12-bound system. The finding supports the mechanism of blocking iNOS dimerization and confirmed our MD results in turn.

**Figure 7:**
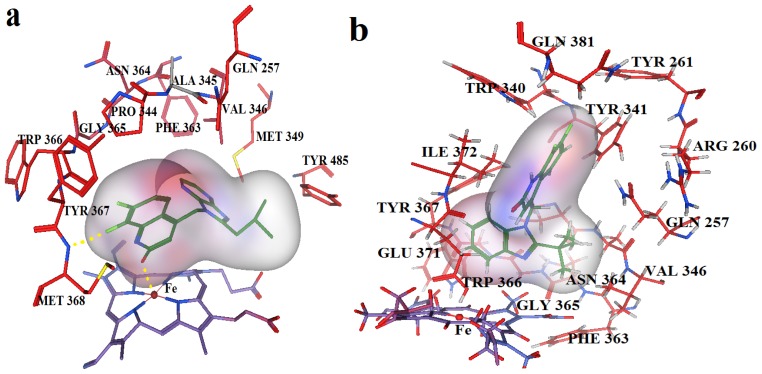
Average conformation of the binding pocket of iNOS (2ORO) and compounds, derived from the last 160 conformations in the last 2 ns MD simulation. Coordination bonds and hydrogen bonds are shown in dashed lines (yellow). (**a**) Compound 34; (**b**) Compound 12.

Average conformations of the binding pocket of iNOS in system-II during the last 2 ns of simulation are depicted in Figure 7. For the 34-bound system, compound 34 occupies the binding pocket. The quinolinone coordinates to the Fe atom, setting the inhibitors into the protein. The substituent of the benzimidazole points back towards the porphyrin, and fills the pocket. The benzimidazole as the linker between the substituent and quinolinone is critical because sufficient flexibility and distance between the two groups are needed to accommodate the bend of the two aromatics and the binding ability of the compounds to the enzyme. Furthermore, the steric amino acid residues around compound 34 at the binding site are shown in Figure 7a. Clearly, no steric amino acid residues appear around positions X_7_ in ring-D (substituent Y), therefore, the substituents at positions X_7_ (conpounds 28, 29, 30, 31) in ring-D improve the activity. However, several crucial amino acid residues are found near some specific positions in the molecules. For example, Trp366, Met368, Tyr367 lies near position 2 (ring-A). It is clear that the aromatic residue Tyr367 can participate in a π–π stacking interaction with ring-A in molecule 34. At the same time, an H-bond with Met368 is formed. Thus, we can conclude that ring-A plays an important role in this binding pocket, due to its ability to form hydrogen-bonds and π-stacking interactions with some residues. Besides, hydrophobic amino acid residues Phe363 and Val346 appear near the X_6_ of ring-D, indicating that molecules with hydrophilic or polar groups (compound 24; pEC_50_ = 1.745) in this area may possess lower binding affinities to iNOS. Moreover, in the same region, the hydrophilic substituent was preferred according to pictorial representation of the contours generated using the QSAR model from Figure 2d. Oversize groups at position 2 of ring-C (compound 5, 6, 7, 8, 9) may be faced with resistance from the hydrophilic residue Tyr485, thereby impairing the activity. On the contrary, compound 12 exhibits lower stability during the 12 ns of the simulation because there is no strong interaction with the Fe atom of the heme. As ring-A and ring-B with hydrophobic groups tend to be away from the heme, the conformation of the protein is altered and the space around the heme becomes more confined. The hydrogen bond acceptor favored region in ring-B is located in the verge of the active site, and the remarkable interactions of this group with some important residues could not be found in the MD results. The ring-C and substituent R_3_ are located at similar positions compared to the MD result of the 34-bound system, but it was observed that most residues in this area such as Asn364, Gly365, Gln257 are not favored with the hydrophobic substituent R_3_ from panel b of Figure 7. This is an appropriate reason to explain why compound 12 exhibits weak inhibitory activity. These results further confirmed our results from the 3D-QSAR model, and revealed that the binding stabilization is consistant with the experimental activities.

## 3. Materials and Methods

### 3.1. Data Sets and Tools

The crystal structure of iNOS with high resolution was retrieved from the protein data bank (PDB code: 2ORO) [15]. Furthermore, the structure was prepared in the following procedures by the Protein Preparation Wizard in the Schrödinger software suite, including adding hydrogens, assigning partial charges using the OPLS_2005 force field and assigning protonation states, and structure minimizing in vacuum [26]. Finally, the cocrystal ligand was removed, and the resulting structure was used as the receptor model in the following studies. Besides, 39 compounds of benzimidazole-quinolinone derivatives with experimental activities are shown in Table 1. The biological activity data was reported in the form of EC_50_ and the EC_50_ values were converted into pEC_50_ using the formula (pEC_50_ = −logEC_50_). The 39 molecules together with their inhibiting activities were taken from the literature [21]. Ligprep module [27] incorporated in Phase was used to convert 2D to 3D structures, and then to proceed with stereoisomer generation, neutralization of charged structures and determination of the most probable ionization state at pH 7.2 ± 0.2.

Phase 3.0 implemented in the Maestro 8.5 software package was used to generate pharmacophore and 3D-QSAR models for iNOS inhibitors [28,29]. All docking studies were performed using Glide 5.0 of the program Maestro 8.5 [30]. The MD simulations were performed with the Desmond software package using the OPLS_2005 force field [31].

### 3.2. 3D-QSAR Studies

The prepared structures were imported to develop pharmacophore model panels of the Phase with their respective biological activity values. The ligands were assigned as actives with a threshold of pEC_50_ > 3 and inactives with a threshold of pEC_50_ < 2.5. Most ligands were flexible, so it was necessary to generate all possible conformations for a ligand in order to increase the chance of discovering the most active conformer. A conformational search generated a set of conformers with a maximum number of 1000 for each ligand using ConfGen method, OPLS_2005 force field and distance-dependent dielectric solvation treatment. To ensure the uniform distribution of structurally different compounds, we calculated the Tanimoto index of 0.19 using the molecule fingerprint in MOE, which showed that the data sets were quite diverse. Then, we divided the data set and made 29 compounds in the training set and 10 compounds in the test set (marked as *). While separating the training set from the test set, we took the distribution of pEC_50_ values in both training and test sets into consideration. Then pharmacophore sites of these compounds were created from a set of six pharmacophore features, including hydrogen bond acceptor (A), hydrogen bond donor (D), hydrophobic group (H), negatively charged group (N), positively charged group (P) and aromatic ring (R). The rules that are applied to map the positions of pharmacophore sites are known as feature definitions, and are represented internally by a set of smarts patterns. Each pharmacophore feature was defined by a set of chemical structure patterns. These pharmacophore sites were characterized by type, location and directionality [22]. Phase searched for common pharmacophores that were common to all actives using a tree-based partitioning technique according to their intersite distances. In the pharmacophore hypotheses scoring process, each pharmacophore and its associated ligand were treated temporarily as a reference in order to assign a score. The hypotheses were ranked according to the following scores: the alignment score, the vector score and a volume score. The predictive power of the QSAR model was validated by the test set compounds. Leave-One-Out techniques were frequently used to validate a QSAR model, and it was useful for assessing the stability of the model. However, internal cross-validated statistics cannot provide a meaningful measure of how the model will actually perform when applied to new molecules. Phase supported external validation, used a true test set whose structures and activities were not considered when QSAR models were developed, so it was far superior to internal cross-validation.

### 3.3. Molecular Docking and Dynamics Simulations

**Figure 8:**
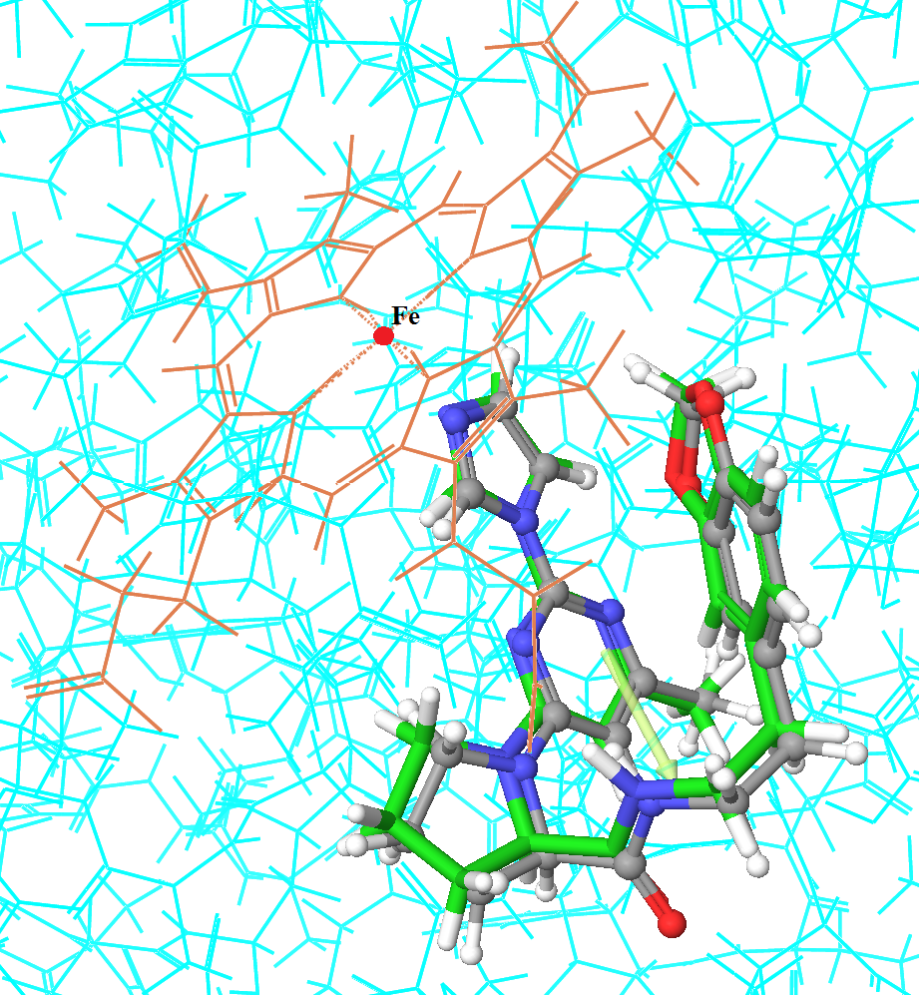
Superposition of co-crystallized BBS-4 (gray) and Glide dock (green).

Molecular docking was performed to further investigate the binding mode between crucial functional groups of benzimidazole-quinolinone derivatives and iNOS. It also aided in understanding the structure-activity relationship revealed by QSAR. Glide was applied to perform the docking studies. Glide approximated a complete systematic search of the conformational, orientational and positional space of the docked ligand, and a series of hierarchical filters was used to search for possible locations of the ligand in the active-site region [30,32]. In this work, grid box was centered on the ligand BBS-4 centroid in the X-ray crystal structure of iNOS (PDB code: 2ORO) and the docking ligand was treated flexibly while the protein was held rigidly in the docking procedure. The RMSD value for heavy atoms of the ligand BBS-4 between the Glide-generated docked pose and the native pose was 0.79 Å (Figure 8). The low RMSD value between the docked pose and the input geometry indicated that the Glide program was able to reproduce the native conformation successfully.

The molecular dynamics of system-I and II were studied using the OPLS_2005 force field in an explicit solvent with the TIP3P model [33] of water within the Desmond software for the MD simulations. The initial coordinates for the MD calculations were taken from the docking experiments. The TIP3P water molecules were then added (the dimensions of each orthorhombic water box were 100 Å × 100 Å × 100 Å, which ensured that the entire surface of each complex was covered by the solvent model, and the systems were neutralized by adding Na^+^ counter ions to balance the net charges of the systems. Before equilibration and long production MD simulations, the systems were minimized and pre-equilibrated using the default relaxation routine implemented in Desmond. For this, the program ran six steps composed of minimizations and short (12 and 24 ps) molecular dynamics simulations to relax the model system before performing the final long simulations. Then, each system was performed for a 12 ns long production MD simulation. The OPLS_2005 force field was used along with the MacroModel module to provide and check the necessary force field parameters for the ligands [34,35]. When MacroModel performs an energy calculation, the program checks the quality of each parameter in use. The use of low quality parameters, especially torsional ones, may result in inaccurate conformational energy differences and geometries. Bond, angle, torsional angle and improper angle checked parameters were listed as high- and medium-quality force field parameters for all ligands studied. During the MD simulations, the equations of motion were integrated with a 2 fs time step in the NVT ensemble. The Shake algorithm was applied to all hydrogen atoms; the van der Waals (VDW) cutoff was set to 9 Å [36]. The temperature was maintained at 300 K, employing the Nose-Hoover thermostat method with a relaxation time of 1 ps [37]. Long-Range electrostatic forces were taken into account by means of the particle-mesh Ewald (PME) approach [38]. Data were collected every 12 ps during the MD runs. Visualization of protein-ligand complexes and MD trajectory analyses were carried out with the VMD software package [39,40]. The equilibration was monitored by examining the stability of the temperature, energy, and the density of the system as well as the RMSD of the backbone atoms.

## 4. Conclusions

In summary, a combined computational approach was applied to give insight into the structural basis and inhibition mechanism for a series of iNOS inhibitors. Molecular docking simulation was used to produce possible binding poses for these compounds to iNOS. Further, MD simulation was performed to confirm the reasonable binding models of iNOS/34 and iNOS/12 complexes. Compared with the MD simulation results, the unaided 3D-QSAR modeling was performed to give further validation of the binding mode and provide a structural framework for understanding the structure-activity relationship of these compounds. Satisfactory agreement between experiment and theory suggested that the atom based QSAR model exhibited good correlation and predictive power. The positive results indicated that the modeling strategies in the present study are most likely to be an encouraging way forward for the elucidation of protein-ligand interaction and in the rational design of novel iNOS inhibitors.

## Figures and Tables

**Table 1:** Structure and activity data of benzimidazole-quinolinone derivatives as inducible Nitric Oxide Synthase (iNOS) inhibitors.

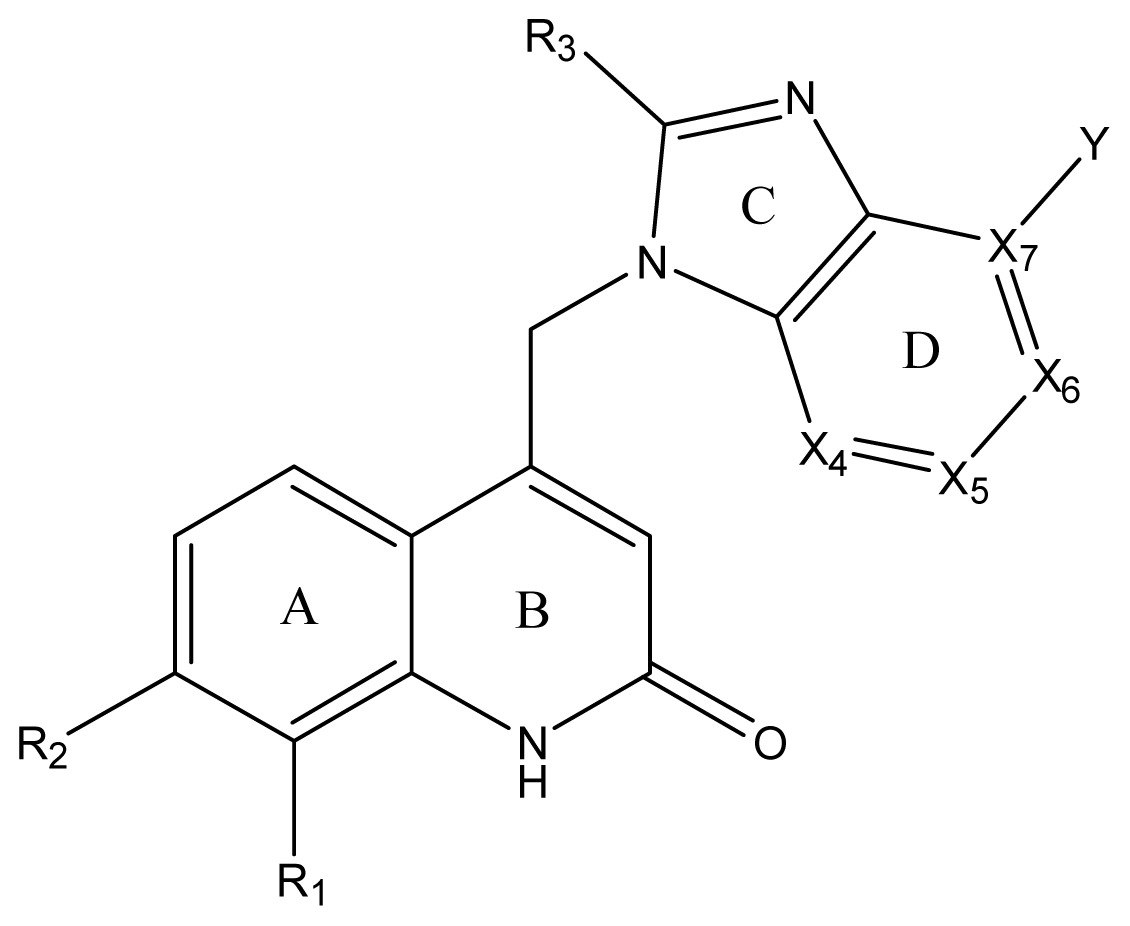												

Compound NO.	X_4_	X_5_	X_6_	X_7_	R_1_	R_2_	Y	R_3_	EC_50_ (mmol/L)	pEC_50_ a (expt.)	pEC_50_ (pred.)	Pharm set
1	C	C	C	C	F	H	H	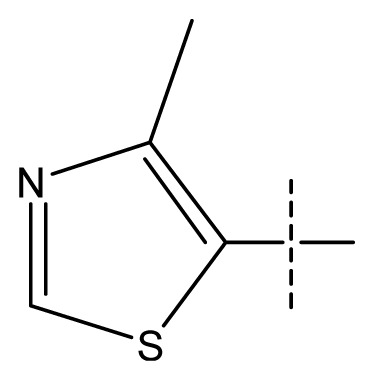	0.0037	2.43	2.29	inactive
2 *	C	C	C	C	F	F	H	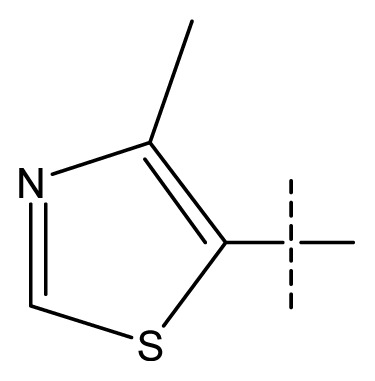	0.0018	2.75	2.61	
3	C	C	C	C	F	F	H	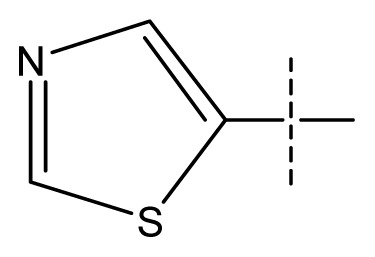	0.0086	2.07	2.04	inactive
4	C	C	C	C	F	F	H	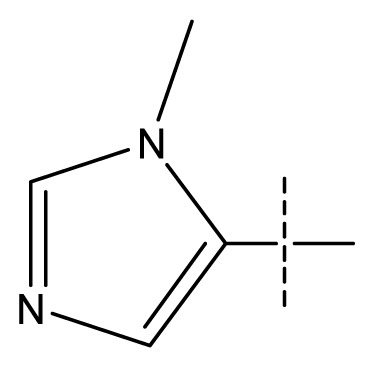	0.0059	2.23	2.09	inactive
5 *	C	C	C	C	F	F	H	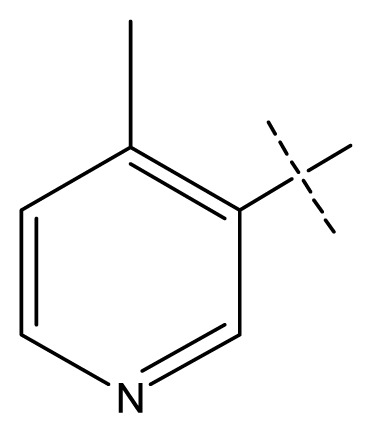	0.012	1.92	2.22	inactive
6	C	C	C	C	F	F	H	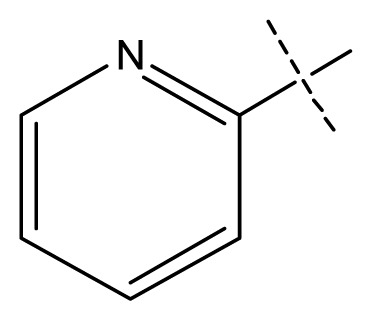	0.018	1.75	1.72	inactive
7	C	C	C	C	F	F	H	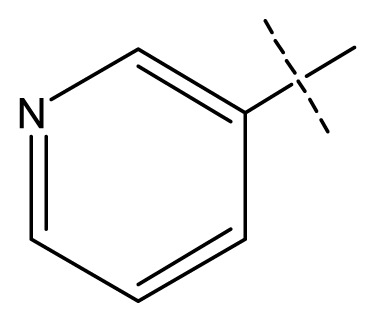	0.0093	2.03	2.11	inactive
8	C	C	C	C	F	F	H	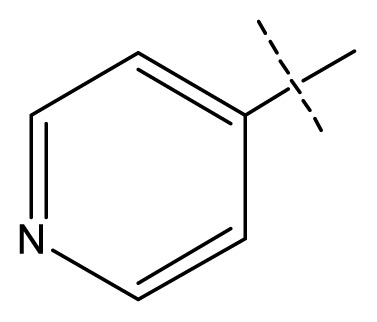	0.0022	2.66	2.55	
9	C	C	C	C	F	F	H	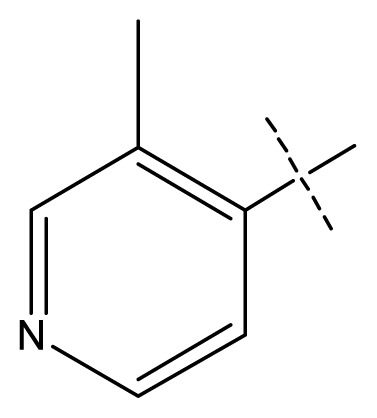	0.00034	3.47	3.40	active
10	C	C	C	C	F	F	H	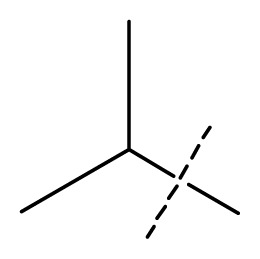	0.00080	3.10	2.79	active
11 *	C	C	C	C	F	F	H	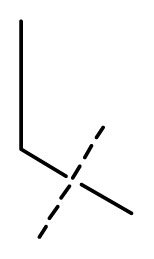	0.0043	2.37	2.49	inactive
12	C	C	C	C	F	F	H	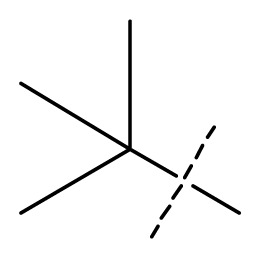	0.048	1.32	1.37	inactive
13	C	C	C	C	F	F	H	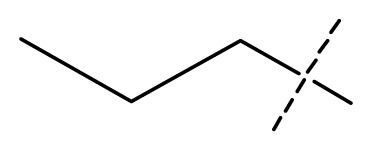	0.00073	3.14	3.35	active
14 *	C	C	C	C	F	F	H	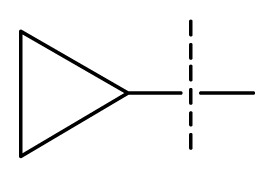	0.0016	2.80	2.83	
15	C	C	C	C	F	F	H	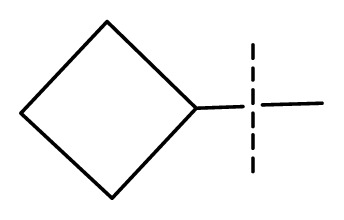	0.00026	3.59	3.21	active
16	C	C	C	C	F	F	H	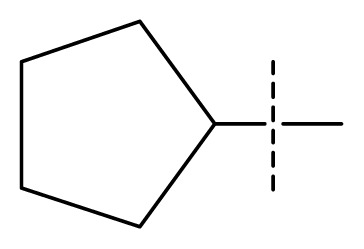	0.00075	3.13	3.12	active
17	C	C	C	C	F	F	H	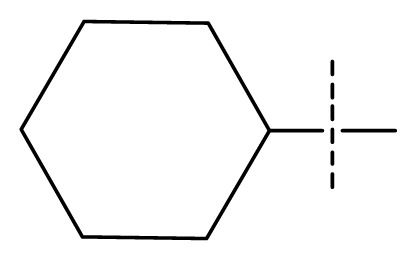	0.0089	2.05	2.33	inactive
18 *	C	C	C	C	F	F	H	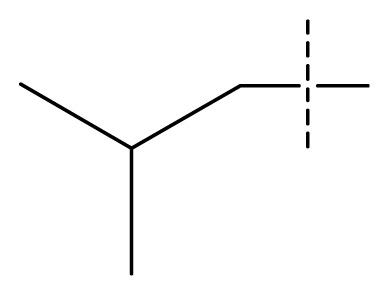	0.00038	3.42	3.05	active
19	C	C	C	C	F	F	H	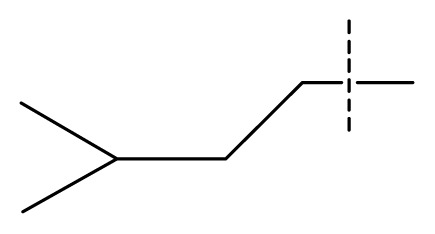	0.00136	2.87	2.84	
20	C	C	C	C	F	F	H	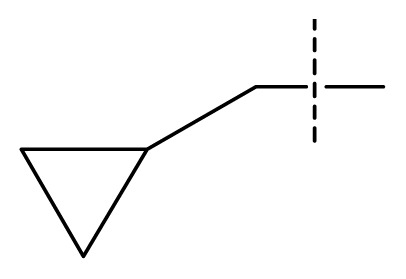	0.00060	3.22	3.50	active
21	C	C	C	C	F	F	H	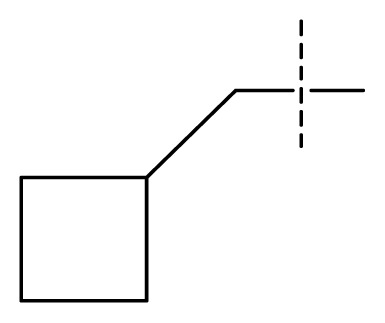	0.00054	3.27	3.24	active
22	C	C	C	C	F	F	H	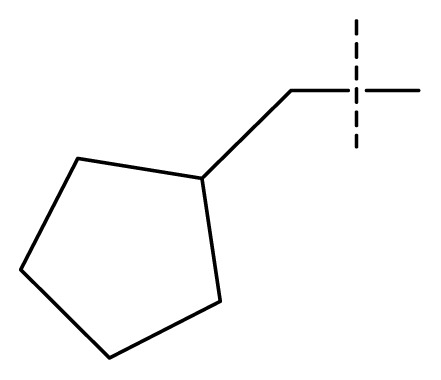	0.00177	2.75	2.76	
23 *	C	C	C	N	F	F		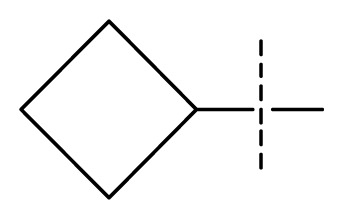	0.00059	3.23	3.01	active
24	C	C	N	C	F	F	H	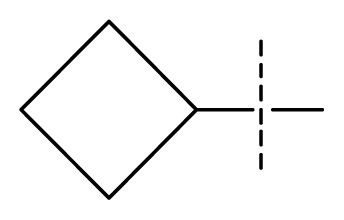	0.018	1.75	2.06	inactive
25 *	C	N	C	C	F	F	H	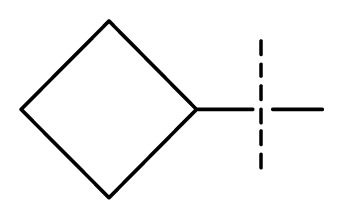	0.00046	3.34	2.90	active
26	N	C	C	C	F	F	H	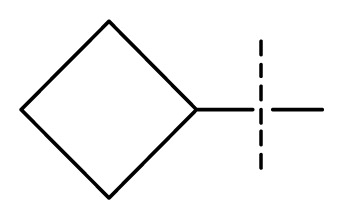	0.00018	3.75	3.69	active
27	N	C	C	C	F	F	Me	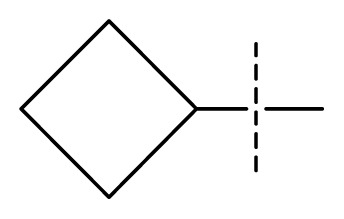	0.00027	3.57	3.56	active
28	N	C	C	C	F	F	Cl	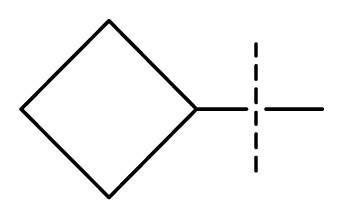	0.00027	3.57	3.61	active
29 *	N	C	C	C	F	F	OMe	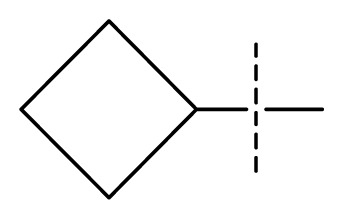	0.00021	3.68	3.78	active
30	N	C	C	C	F	F	NMe_2_	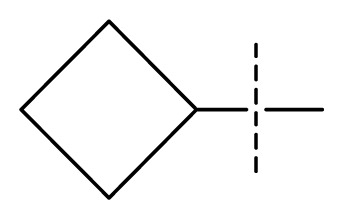	0.00017	3.77	3.83	active
31 *	N	C	C	C	F	F	CF_3_	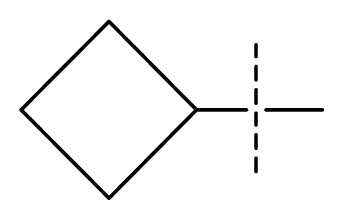	0.00015	3.82	3.69	active
32 *	N	C	C	N	F	F		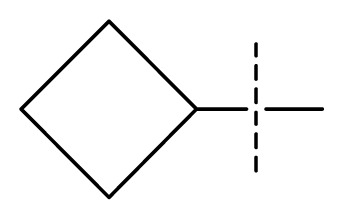	0.000091	4.04	3.70	active
33	N	C	C	N	F	F		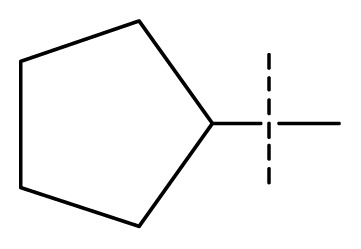	0.00028	3.55	3.67	active
34	N	C	C	N	F	F		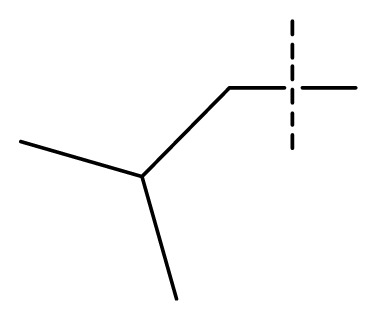	0.000088	4.06	3.70	active
35	N	C	C	N	F	F		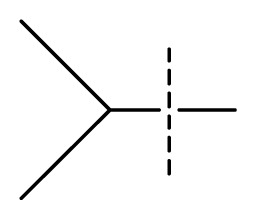	0.00085	3.07	3.12	active
36	N	C	C	N	F	F		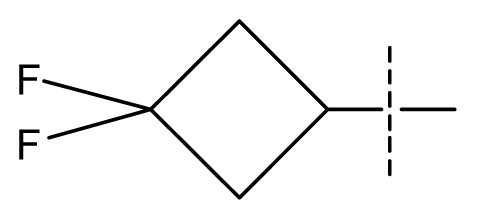	0.00012	3.92	3.58	active
37	N	C	C	N	F	H		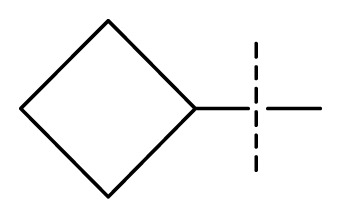	0.00019	3.72	3.88	active
38	N	C	C	N	F	H		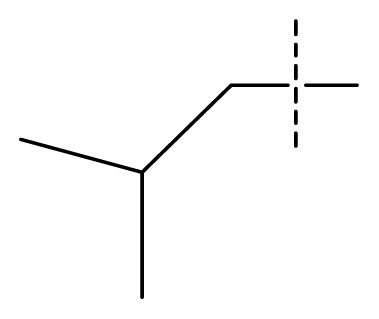	0.00029	3.54	3.68	active
39	N	C	C	N	F	H		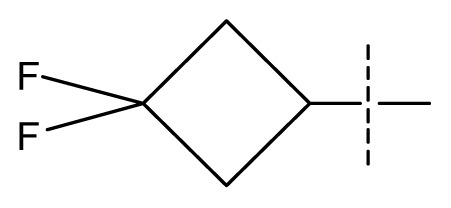	0.00022	3.66	3.58	active

a Each pEC_50_ was calculated based on the reference 21. *N*-(3-(Aminomethyl)benzyl) acetamidine (1400 W), as one of the most selective inhibitors of purified human iNOS reported to date, was chosen as a positive control in the iNOS activity inhibition assay, and the EC_50_ of 1400 W was 0.1 mmol/L [21];

* The test set compounds are marked by an asterisk.
